# Detection of Active Wood-Boring Insect Larvae Using Acoustic Emission Measurements: Principles, Experimental Validation, and Practical Applications

**DOI:** 10.3390/s26154672

**Published:** 2026-07-23

**Authors:** Lisa Anna Limmer, Burkhard Plinke, Constanze Messal, Stephan Biebl

**Affiliations:** 1 MICOR Gesellschaft für Mikrobielle Prozesse und Materialkunde mbH Rostock, 18069 Rostock, Germany; constanze.messal@hawk.de; 2 Independent Researcher, Formerly WKI Braunschweig, Fraunhofer Institute for Wood Research, 38108 Braunschweig, Germany; 3 Faculty of Architecture, Engineering and Conservation, HAWK Hildesheim, University of Applied Sciences and Arts, 31134 Hildesheim, Germany; 4 Ingenieurbüro für Holzschutz, 83671 Benediktbeuern, Germany; info@holzwurmfluesterer.de

**Keywords:** acoustic emission, bioacoustics, wood-boring insects, non-destructive testing, structure-borne sound, *Hylotrupes bajulus*, *Anobium punctatum*, cultural heritage, IADS, insect activity detection

## Abstract

**Highlights:**

**What are the main findings?**

- Wood-boring insect larvae activity can be reliably detected using structure-borne acoustic emission measurements.
- The technology has been verified in a broad range of lab and field conditions.

**What are the implications of the main findings?**

- A portable Insect Activity Detection System is available and ready to use.
- However, expertise in infestation assessment remains essential.

**Abstract:**

The larvae of wood-boring insects often cause damage to wooden buildings, furniture, and objects of cultural heritage. A fundamental practical challenge in assessment and conservation is reliably distinguishing between active and inactive infestations—a distinction that conventional visual inspection methods cannot always resolve with confidence. We present a selection of experiments for the implementation of acoustic emission (AE) measurement methods for these inspections, spanning from highly controlled laboratory settings to practical field tests. An overview explains the physical principles of structure-borne sound emission by feeding larvae and the AE sensors and other instrumentation used (AMSY-6 stationary system and the single-channel Insect Activity Detection System, IADS). Laboratory experiments with standardized small specimens containing single larvae of *Hylotrupes bajulus* are described in detail, examining the influence of temperature, sensor coupling method, and sensor-to-larva distance on detection reliability. Verification experiments on large structural timber with unknown infestation levels demonstrated the practical applicability of the method and enabled qualitative localization of larval activity. Field applications in historic buildings, churches, and cultural heritage objects are presented and discussed. The results confirm that AE sensors can reliably detect larval activity of *H. bajulus* in standardized specimens at signal-to-noise ratios clearly distinguishable from negative controls. Sensor sensitivity decreases significantly with distance, with reliable detection up to approximately 30 cm under the tested conditions. Although interpretive expertise in wood biology, infestation assessment, and conservation expertise remains essential, the results indicate that AE sensors can indeed be used to detect larval activity inside wood in a non-destructive way in a broad range of field and laboratory applications and across a range of conditions.

## 1. Introduction

### 1.1. Background: Problems Caused by Wood-Boring Insects

**Figure 1:**
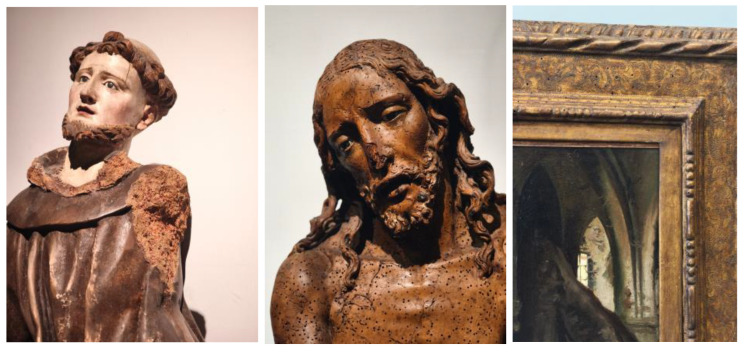
Representative examples of damage to wooden cultural heritage objects caused by wood-boring insects. (**Left**): polychrome wooden sculpture showing structural material loss caused by insect galleries (Monastero di Santa Chiara, Naples). (**Center**): wooden figure with extensive emergence hole pattern consistent with *Anobium punctatum* or similar small boring species. (**Right**): detail of an ornate picture frame showing emergence holes and frass deposits (Alte Nationalgalerie, Berlin). Photos: C. Messal.

Wood-boring insects pose a persistent threat to wooden buildings, structural timber, furniture, and cultural artifacts worldwide. The larvae of xylophagous (wood-eating) species cause damage by excavating feeding tunnels through the wood matrix, progressively destroying both structural integrity and historical substance (Figure 1). Among the species of greatest practical concern in Central Europe are the old house borer (*Hylotrupes bajulus* L.; Cerambycidae), which attacks softwood and has historically caused widespread damage to roof structures and load-bearing timbers [1,2]; the common furniture beetle (*Anobium punctatum* De Geer; Anobiidae), which infests both hardwood and softwood and is among the most frequently encountered species in furniture and cultural heritage objects [3]; the brown powderpost beetle (*Lyctus brunneus* Stephens; Lyctidae), which attacks the sapwood of ring-porous hardwoods; and the deathwatch beetle (*Xestobium rufovillosum* De Geer; Anobiidae), which favors partially decayed (mold infested) hardwood in modern and historic structures. Additionally, the increasing global movement of goods and the effects of climate change are raising the risk of invasive species becoming established in previously unaffected regions—with the Asian longhorned beetle (*Anoplophora glabripennis* Motschulsky; Cerambycidae) being a particularly notable example, with repeated introductions into Central Europe and North America via infested packaging timber [4].

### 1.2. Established Infestation Evaluation Methods and Their Limitations

In practical assessment, as well as in conservation and restoration contexts, a fundamental diagnostic challenge is determining whether a visible infestation is still active or has already died. Visual indicators such as emergence holes, frass (bore dust), and feeding galleries are unreliable for this purpose due to the following reasons: old frass may be redistributed mechanically [5,6]; the secondary use of abandoned galleries by non-xylophagous species such as *Corynetes coeruleus* (Fab.) (both of these reasons can lead to false conclusions); and the internal larval stages—eggs, early-instar larvae, and pupae—leave no surface trace whatsoever. The established method of sealing suspected areas with Japanese tissue paper or thin foil and monitoring over months to years for the appearance of new emergence holes is reliable in principle, but is impractical for many objects, incompatible with some conservation requirements, and permits destructive activity to continue uninterrupted throughout the monitoring period [2].

### 1.3. Technical Methods for Infestation Detection

Several instrumental methods have been proposed to supplement or replace visual inspection. Radiographic imaging (X-ray) can map feeding galleries and, under favorable conditions, distinguish living from dead larvae [7]; however, it requires laboratory access, complete volumetric scanning of the suspect object, and three-dimensional image reconstruction, making targeted field application to large timber elements impractical. Additionally, due to the health hazard X-rays pose, equipment must be operated by specialists. Computed tomography offers excellent spatial resolution but remains confined to laboratory settings and is disproportionately costly for routine infestation assessment. Infrared (IR) thermography is also of very limited practical utility for detecting live insects in solid wood [8]. Since insect larvae, as ectotherms, usually do not generate relevant amounts of metabolic heat, the thermal excess relative to the surrounding wood and ambient environment is generally too small to be resolved reliably using thermographic imaging under realistic conditions. This limitation is compounded by wood’s low thermal conductivity and varying moisture contents, which further complicate measurements and result in IR thermography generally being unsuited to detecting active larvae in wood.

An alternative approach that avoids these constraints is the measurement of structure-borne sound signals generated by larval feeding activity. When larvae cut through wood fibers under mechanical tension with their mandibles, they generate transient acoustic events (crack-like impulses) that propagate through the wood as structure-borne sound (elastic waves). Large larvae of species such as *H. bajulus* produce feeding sounds audible to the human ear under quiet conditions; smaller larvae or more distant sources require electronic amplification and sensitive contact sensors. The acoustic emission (AE) technique, established in materials testing and structural health monitoring as a method for passive detection of transient elastic energy releases [9,10], provides a well-characterized framework for this application. AE sensors measure these transient events in the frequency range above 20 kHz, where ambient airborne noise is effectively excluded, allowing sensitive detection even in environments with moderate background sound levels.

The use of acoustic methods for detecting insect activity in wood has a history spanning nearly a century [11]. Emerson and Simpson first reported amplified detection of movement and communication signals from termites in 1929 using repurposed parts of a telephone transmitter [12]. An early commercial listening device specifically for *H. bajulus* detection—the “Bajuphon”—was marketed in Germany in the early 1950s [13]. A systematic scientific investigation of larval feeding acoustics using contact sensors was achieved by Pallaske, who was able to quantify *H. bajulus* larval activity rhythms and demonstrated that electronic recordings could document behavioral patterns non-destructively [14,15]. Pallaske also already proposed this as a possible detection method [16]. A few decades later, Plinke used piezoelectric AE sensors for wood-boring insect detection and established that signals useful for detection were concentrated predominantly below 10 kHz, with a bandpass filter of 500 Hz … 5 kHz effective for ambient noise rejection [17]. Subsequent studies demonstrated detection of termite activity [18] and of multiple dry-wood-boring species with commercial AE instrumentation [19,20]. Working with the “Woodworm Detector”, a commercial device from the USA (which was discontinued years ago and is therefore no longer available), and other piezoelectric sensors, Creemers was able to establish practical parameters including temperature thresholds [19] and the effect of sensor coupling method on signal transmission.

More recent work has investigated signal-based localization [21], the relationship between larval body mass and detectable AE energy [22], the detectability of newly hatched larvae [23], and machine learning approaches to signal classification [24]. However, the systematic integration of laboratory validation with field application protocols, across multiple species and coupling conditions, and within a single methodological framework suitable for practical conservation and wood protection contexts remains incomplete in the published literature.

### 1.4. Objective of This Work

This article addresses this gap through a systematic investigation of available AE equipment for detecting larvae activity focused on the following relevant aspects:

- The physical and instrumentation principles underlying AE-based larval detection;
- The results from a series of controlled laboratory experiments examining the effects of temperature, sensor coupling method, and sensor-to-source distance on detection performance;
- Verification on large structural timber with unknown infestation levels using parallel measurements with two instrument types;
- Documented field applications in historic buildings and on cultural heritage objects.

The data reported in Section 3.1, Section 3.2 and Section 3.3 originate from a Master’s thesis [25] and have been partially reported in conference proceedings and a reviewed journal article [26]. The large-sample verification in Section 3.4 is more thoroughly described in the final report of the project “InsectDetect” [27] and in a conference paper [28]. The field applications in Section 3.5 include previously unpublished material. The present article constitutes an extended and integrated synthesis of these sources and aims to showcase the systematic evaluation of AE sensors for an application they were not originally designed for yet continue to prove effective and valuable.

## 2. Materials and Methods

### 2.1. Acoustic Emission Measurement Principle

Acoustic emission (AE) refers to the phenomenon whereby transient elastic waves are generated within a material as a result of rapid local energy release—typically associated with crack propagation, phase transformation, [9,10] or, in the present context, the mechanical cutting of wood fibers by feeding insect larvae. These waves propagate through the solid as structure-borne sound and can be detected at the material surface by piezoelectric sensors coupled to the substrate.

An important operational consideration is signal attenuation: AE waves are attenuated as they propagate through wood, with attenuation significantly greater across the grain than in the fiber direction [10]. Wood’s acoustic anisotropy means that the effective detection range is directionally dependent, and joints, glue lines, or variations in wood density such as for early wood/late wood reduce signal transmission. These factors are directly relevant to the practical application of AE in wood protection and conservation.

**Figure 2:**
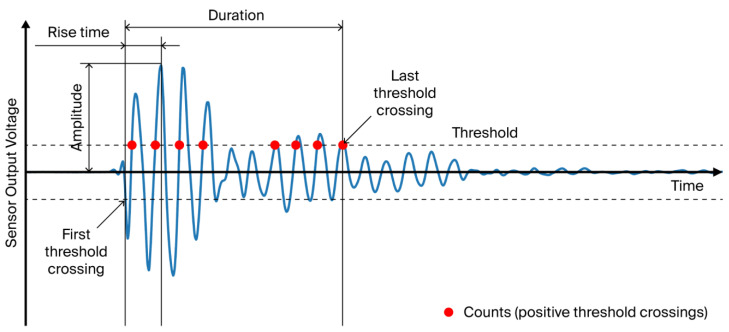
Schematic representation of a single acoustic emission hit waveform with characteristic parameters: threshold, peak amplitude (dB), counts (threshold crossings, marked as dots), and hit duration. Courtesy of Vallen Systeme.

The important parameters of AE signals are amplitude, pulse rate, frequency spectrum, and correlations between these parameters and other boundary conditions, e.g., a mechanical load during destructive testing or the material temperature. AE signals can be characterized by the parameters shown in Figure 2: The signal, i.e., the voltage supplied by the sensor, is recorded as soon as a certain amplitude (“threshold”) is exceeded for the first time. From this point onwards, the voltage curve of the “hit” is recorded by a transient recorder until it falls below the threshold again. The amount of threshold crossings (red dots in Figure 2) and the maximum amplitude within the duration are sufficient characteristics for a “hit” for the purpose of larvae activity detection.

The result of an AE measurement over a longer period of time (minutes to several days) initially comprises only the characteristics of the registered hits, shown here in the form of a data record per hit, which is stored together with a time stamp in an SQL database. A known limitation with wood particle materials and solid wood is that sound propagation occurs with different attenuation depending on the composition and is usually anisotropic, i.e., with direction-dependent velocities, so that the geometry and nature of the sample must also be taken into account when evaluating the data. As many measurement channels as possible should be available. Locating sound sources in wood is therefore only possible under favorable conditions, e.g., in homogeneous solid wood with a known geometry and sound propagation in the grain direction.

The frequency spectrum within the measurement interval was always very similar in our tests, regardless of the signal source. For the subsequent evaluation, the features “peak amplitude” (maximum of the signal) and “counts” (number of times the threshold value was exceeded in the respective measurement interval) therefore proved to be sufficient and useful. The software records the data as hits with the feature vector and time stamp in an SQL database. The data can be graphically processed, filtered, and displayed in real time during the measurement process. However, the evaluation—e.g., whether larval activity has been recorded—must be carried out by the user.

### 2.2. Equipment and Data Evaluation

**Figure 3:**
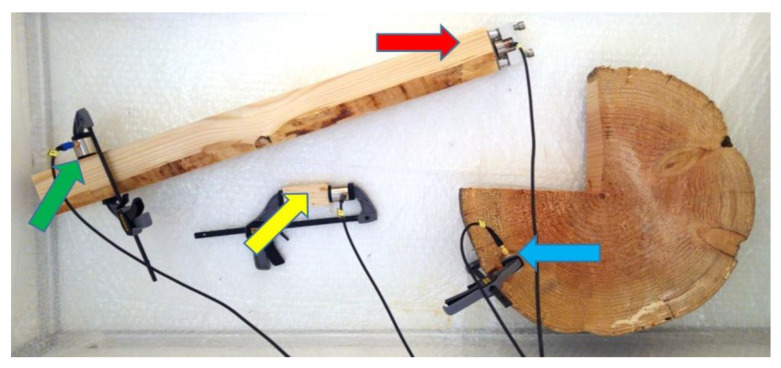
Sample composition for measuring method demonstration: Channel 1 (left, green): sensor clamped to the side of the left end of the squared timber near the larva (old house borer). Channel 2 (top, red): sensor in the direction of the grain on the squared timber approx. 40 cm away, coupled with magnetic holder. Channel 3 (bottom center, yellow): sensor clamped axially to the small sample with larva (sapwood beetle). Channel 4 (bottom right, blue): sensor clamped in fiber direction to a tree slice with larva (old house borer). Photo: B. Plinke.

The sensors comprise robust steel cylinders with a ceramic piezoelectric element on the sensitive front surface. They are available for different frequency ranges; a sensor frequency range above 20 kHz is required for satisfactory suppression of audible background noise. They can be clamped onto samples with different geometries using commercially available adjustable one-hand clamps of a suitable size. For a planed wood surface, dry coupling and adjusting the clamp to a pressure of approx. 5 … 10 N/cm^2^ is sufficient. For rough surfaces, if necessary, an additional magnetic plate (Figure 3) can be used. However, it should be noted that the magnetic plate requires screws to be driven into the wood, in which case the coupling method is no longer non-destructive and can only be applied in cases where this is not an issue. Therefore, this coupling method is only suitable for sections of logs with bark, but not for art objects. Adequate coupling can be checked using the pencil lead test (see Section 2.2).

**Figure 4:**
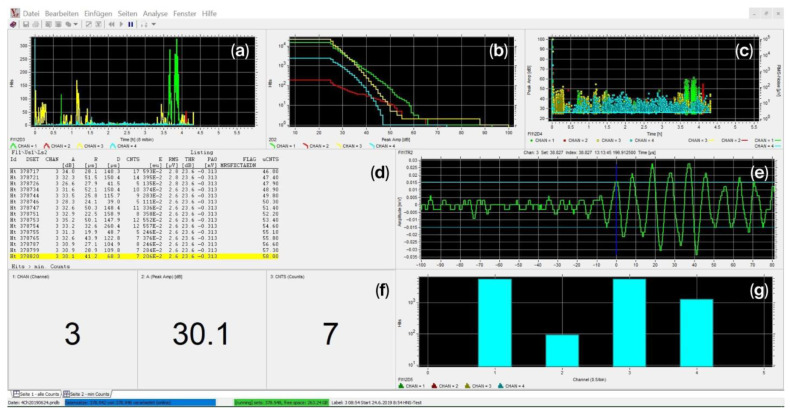
Processed measurement results for the samples in Figure 3; color coding as in Figure 3: pulse rate (hits per 10 s, linear)/measurement duration (**a**); number of hits (log.)/peak amplitude (**b**); peak amplitude (dB)/measurement duration (**c**); listing of hit characteristics (minimum 5 counts) (**d**); signal curve of last hit (**e**); channel number (CHAN), peak amplitude, counts of last hit (CNTS) (**f**); total hits (logarithmic) for all channels, accumulated over the measurement interval (**g**). Graph: B. Plinke.

Figure 4 demonstrates the quantitative results from the four measurement channels created using Vallen VisualAE (Software Release R2019). Only the hits with a minimum number of (in this example) 5 threshold value crossings (counts) are taken into account. Additionally, the results are displayed as the course of the pulse rate over the measurement duration, and as a bar chart comparing the activities of all channels over the entire measurement duration.

Pre-processing the AE signals before recording ensures that only hits typical of larval activity are registered: the sensitivity of the sensor above frequencies of 20 kHz largely excludes audible background noise, and because only pulses with at least 3 or 5 threshold crossings (counts) are taken into account, only those above a minimum duration are used to determine the pulse rate. This requires lower data storage requirements and computing time and enables live evaluation, unlike the approach of first recording all signals and evaluating them later [29,30]. It is also possible in principle to extract further characteristics of AE signals and use these to make more accurate predictions about the positions and population of larvae [31]. However, such methods require comprehensive training on the particular combination of sample composition and geometry, larval species, and environmental variables such as temperature. The method described here is intended to be a simple method for rapid testing that is less demanding in terms of data handling, allows live display of larval activity, and is applicable across a wide range of use cases.

The pulse rate is the most important evaluation criterion for determining whether a sample is active or not. A pulse rate that is permanently or temporarily above one hit per 10 s (360 hits/h) already indicates activity. However, larvae are not constantly active; if activity is to be ruled out, the measurement time should be 24 h if possible.

The stationary AMSY-6 configuration [27] (Manufacturer: Vallen Systeme GmbH, Wolfsratshausen, Germany) consists of one or more AE sensors (Type VS45-H) and preamplifiers (Type AEP5) per channel, one rack with 4 configurable signal processing ASIP-2/A, and a PC for live display and data evaluation. The system is scalable: in the research project InsectDetect and the investigation on large samples (Section 3.4), we used 4 sensors, but additional sensors and channels can be added. With the AMSY-6 configuration, cables between the preamplifier and computer were 10 m long (the technical limit is 600 m), permitting larger components or objects in a quarantine chamber or climate chamber to be measured over periods of several weeks. The frequency filters in the signal processor units were set to a lower cut-off frequency of 80 kHz, and the sampling rate was 2.5 MHz. At the set gain, noise signals in zero samples resulted in hits with a peak amplitude below approximately 23 dB, while signals in the Hsu-Nielsen test yielded a value above 85 dB. Therefore, the lower threshold for detecting a hit in this sensor configuration was normally set to 24 dB to be sure to measure only hits with a peak amplitude above the noise level. In environments with background noise, it has been shown that the sensor frequency range effectively suppresses interference. These settings were used for the investigations described in Section 3.4 and Section 3.6.

**Figure 5:**
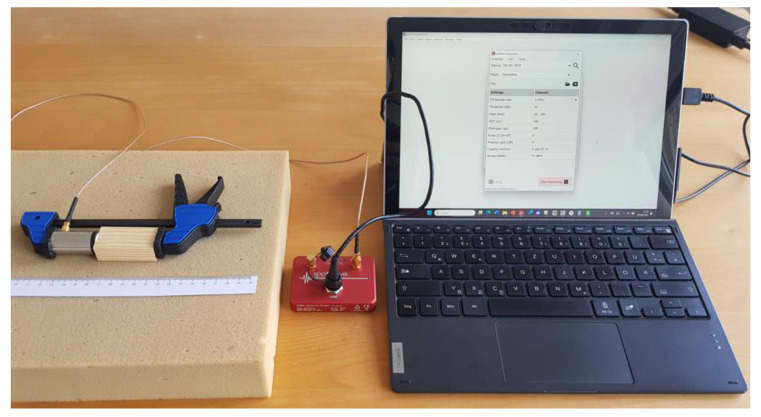
The main components of the IADS configuration coupled to a standardized wood specimen (50 × 25 × 15 mm^3^) using an adjustable one-hand clamp, as used in small-sample laboratory experiments. (Source: Ref. [25]).

The spotWave configuration (Manufacturer: Vallen) has been designed for a mobile single-channel application, using a small housing for amplification and signal processing and a laptop or tablet for recording and evaluation (Figure 5). A similar sensor type (Type VS30-V, Manufacturer: Vallen) with a modified frequency range and sensitivity characteristics, but compatible with data structures of the evaluation software VisualAE is very suitable. The sensor and signal processing differ from the stationary configuration with the AMSY-6 system in terms of frequency response and sensitivity. If the frequency range of the analogue pass band filter is set to 20 … 100 kHz, the lower detection threshold to 34 dB, and the sampling rate to 1 MHz, comparable hit characteristics and measurement results can be achieved. These settings were used for the investigations described in Section 2.3, Section 2.4, Section 3.1, Section 3.2 and Section 3.3 and Section 3.5. This mobile configuration is now in operational use as the Insect Activity Detection System (IADS) [32]. Experimental verifications and field applications of the AE measurement method using the stationary AMSY-6 system and the IADS are described in the following sections.

### 2.3. Specimen Preparation and Experimental Design

All small-sample experiments used standardized wooden test specimens of uniform dimensions (50 × 25 × 15 mm^3^). This specimen size was chosen to correspond to standardized test specimens used in biocide efficacy testing at accredited testing institutions (e.g., MPA Eberswalde), facilitating comparability with toxicological test series and ensuring a supply of reproducible infested specimens. Specimens were prepared by introducing a single larva into a pre-bored cavity of appropriate dimensions before sealing; in all cases, the species, instar, and, in case of *Hylotrupes bajulus,* approximate sizes of the enclosed larva were known. Negative control specimens of identical dimensions but without larvae were prepared in parallel from the same wood lots. Infested *H. bajulus* specimens were stored at 10 °C between measurements to limit larval development and standardize metabolic state. The larvae of *Hylotrupes bajulus* were at various stages of development, with weights ranging from 50 mg to 500 mg. Meanwhile, it is very difficult to determine the weight of an *Anobium punctatum* larvae. The aim of the investigations was only to identify larval activity. Correlations between the weights of the larvae and the measured pulse rates were not examined in detail.

Prior to each measurement, the quality of the acoustic coupling between sensor and specimen was verified using the Hsu–Nielsen source (pencil lead break, PLB) test [9], in which a 0.5 mm mechanical pencil lead is pressed lightly against the specimen surface and broken by tilting the pencil. This produces a controlled, reproducible AE signal physically analogous to a wood fiber cracking, with an amplitude approximately 30 dB higher than typical larval hits. PLB tests were performed at the beginning and end of each measurement interval; results confirming adequate coupling were a precondition for inclusion of the data in analysis. PLB events are identifiable in the data stream by their characteristic high amplitude and are excluded from hit rate calculations.

### 2.4. Data Processing and Hit Rate Calculation

Raw AE data were processed following the procedure described by Plinke [27] and adopted in subsequent IADS studies [26]. The total number of recorded hits within a measurement interval, after exclusion of PLB events, was normalized to hits per hour (hits/h) to enable comparison across measurement intervals of differing duration.

For some data, a secondary filter was applied within the Vallen VisualAE software as recommended by B. Plinke (pers. comm.). The filter excluded all hits with less than three counts per hit, the underlying hypothesis being that this further reduces noise. Since optimal filter settings are still under investigation, for some experiments, both unfiltered hit rates (without the mentioned secondary filter; denoted “U”) and filtered hit rates (hits additionally subjected to the secondary filter and therefore all hits with less than three counts per hit removed; denoted “F”) are reported throughout this article. This dual reporting ensures transparency and reproducibility, and allows readers to assess the sensitivity of results to the filter choice.

**Figure 6:**
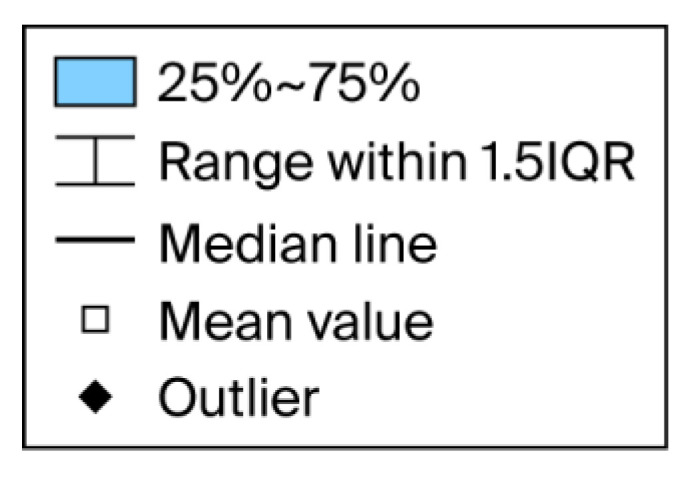
Legend for the boxplots created in Origin 2021. 1.5IQR means 1.5 × Interquartile range.

For the evaluation of the results of the respective experiments, outlier tests according to Grubbs were performed against a single outlier (*p* ≤ 0.05) to identify statistical outliers if enough values of a category were present. For statistical analysis, a one-way ANOVA was performed, followed by a Tukey post hoc test for pairwise comparison, where *p* < 0.05 was considered statistically significant (software: Origin 2021). The results were plotted as boxplots using Origin 2021 (Figure 6).

## 3. Results

### 3.1. Temperature Effect on AE Detection of H. bajulus Larvae

The literature establishes 10 °C as a provisional lower temperature threshold for productive AE measurements of wood-boring insect larvae, with hit rates declining sharply below this temperature [19]. To determine whether a warm-up period was required before measurement of specimens stored at 10 °C, and to quantify the temperature dependence of larval activity, *H. bajulus* specimens (*n* = 20 infested, *n* = 1 negative control, pine) were measured during two phases: First, immediately after removal from 10 °C cold storage (“cold measurement”), and a second time again after several hours of acclimatization to room temperature (varying temperatures, average of 26.36 °C (rounded); “warm measurement”).

**Figure 7:**
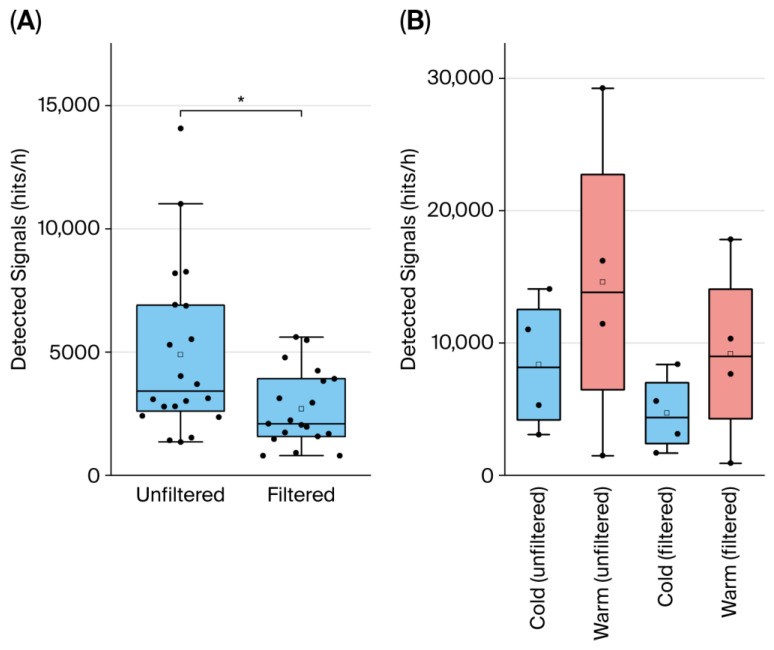
(**A**): Temperature experiment results for *Hylotrupes bajulus* specimens (H.1–H.20) measured directly after storage at 10 °C. Representation of the measured signals in hits/h. Explanation of the box plot symbols see Figure 6. The * and bar symbol on top mark experiments with significant different results. Outlier test according to Grubbs was not applied to measurements with negative sample (as there were too few values). (**B**): Results for specimens measured after several hours of storage at room temperature (red, warm) together with the values of the corresponding specimens previously measured directly after storage at 10 °C (blue, cold). For the diagram (**B**), showing the “cold/warm” comparison, only four samples were measured under “cold” as well as under “warm” conditions; therefore, the boxplots show only four data points in “blue” and “red” each. (Source: Figure 26 in Ref. [25]).

Acoustic emission hits were recorded in all measurements involving infested specimens. Hit rates under warm conditions consistently exceeded those recorded immediately after cold storage for each matched specimen (Figure 7B). Despite this consistent directional trend, no statistically significant difference between cold and warm conditions was detected across the sample population, likely attributable to the high variability in individual larval activity (Figure 7A) and the moderate sample size. The single negative control specimen yielded 0 hits/h under both unfiltered and filtered analysis, consistent with the absence of a signal source.

These results confirm that reliable AE detection of *H. bajulus* larvae is achievable directly after removal from 10 °C storage, without a mandatory warm-up phase. The consistently higher hit rates after room-temperature acclimatization indicate that larval activity is positively associated with ambient (and hence internal wood) temperature, consistent with their ectothermic physiology and prior reports [19,33]. Given the insulating properties of wood, acclimatization time should be scaled to specimen dimensions—for small specimens (50 × 25 × 15 mm^3^), the time required was short; for larger structural elements, substantially longer acclimatization times may be needed. Pending more comprehensive investigations, a lower measurement threshold of 10 °C is provisionally recommended, consistent with the literature [19].

### 3.2. Effect of Sensor Coupling Method on AE Detection

**Figure 8:**
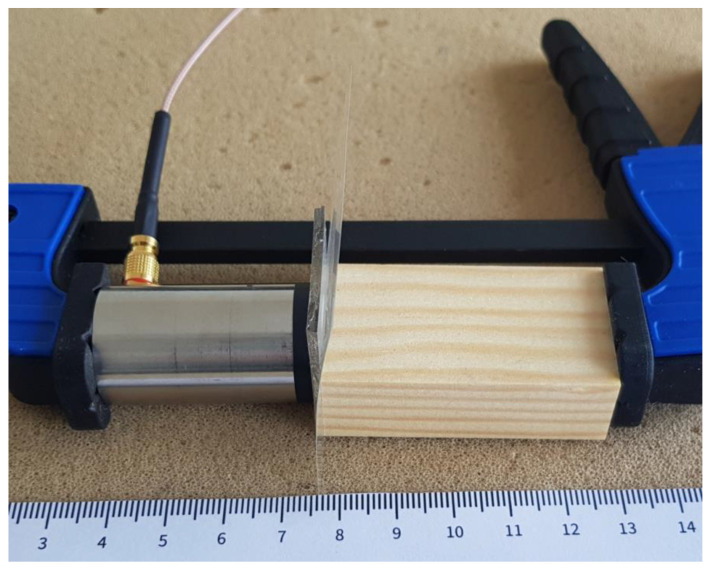
Coupling of the sensor via clamp to pine specimen with 3 mm silicone foam and thick Hostaphan^®^ PET film as intermediate layers. The larva was initially placed on the right-hand side of the specimen and was situated approximately between 110 and 120 mm along the scale. Photo: L. Limmer.

Reliable signal transmission between the sensor and wood surface is a prerequisite for meaningful AE measurements. In conservation and heritage applications, coupling methods must be non-destructive with respect to the substrate surface. Five coupling methods were evaluated on *H. bajulus* specimens in pine (*n* = 20 infested, H.1–H.20; *n* = 2 negative controls, P.1 and P.5). Each specimen was measured for 15 min under each coupling method (example of one method in Figure 8). The five tested methods were as follows:

1. Clamp (direct clamp, no intermediate material; reference condition);
2. Sticky dot (acrylic adhesive dot, removable);
3. Nano tape (reversible nano-adhesive tape);
4. Cardboard (clamp with museum conservation board and thin Hostaphan^®^ PET film (Manufacturer: Mitsubishi Polyester Film GmbH, Wiesbaden) as intermediate layers);
5. Silicone pad (clamp with 3 mm silicone foam and thick Hostaphan^®^ PET film as intermediate layers).

**Figure 9:**
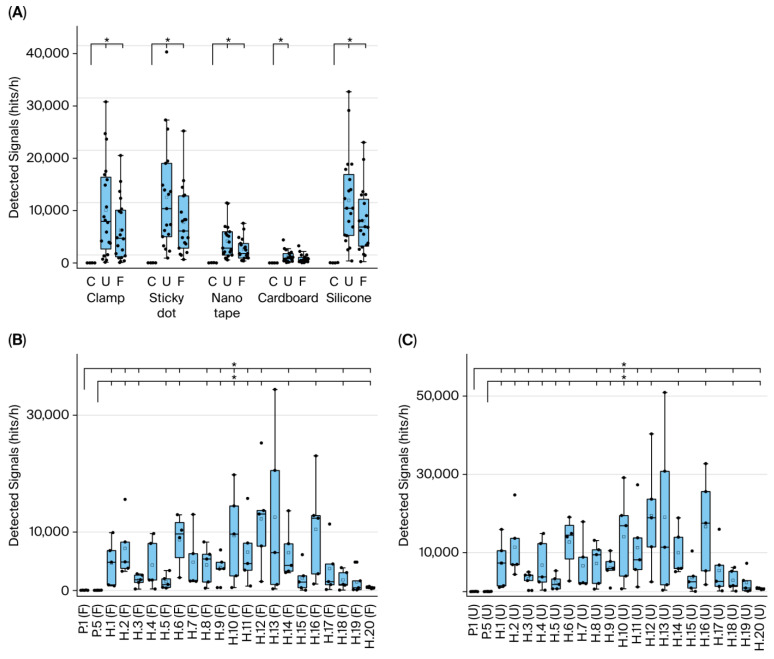
Coupling test results for the *Hylotrupes bajulus* specimens in coniferous wood (H.1–H.20) and two coniferous wood negative specimens (P.1; P.5) with five different coupling methods. Representation of the measured signals in hits/h. Explanation of the box plot symbols see Figure 6. The * and bar symbol on top mark experiments with significant different results. Outlier test according to Grubbs was not applied to measurements with negative samples (as there were too few values). (**A**): Measured hits/h for the different coupling methods. C = Negative control samples; U = Unfiltered; F = Filtered. (**B**): Coupling test measurements broken down according to the individual test specimens, filtered (F). (**C**): Coupling test measurements broken down according to the individual test specimens, unfiltered (U). (Source: Figure 29 in Ref. [25]).

According to PLB tests before and after each measurement interval, all five methods achieved successful signal coupling. Across all coupling methods, a statistically significant difference in hit rate between infested specimens and negative controls was observed (Figure 9), demonstrating successful larval activity detection under each condition. To illustrate the magnitude of the signal contrast, the negative control P.5 registered a single hit during a 15-min measurement interval; specimen H.16 (one *H. bajulus* larva) registered 4380 hits in the same interval under the clamp coupling condition.

Hit rates were lowest for the cardboard/thin Hostaphan^®^ method (coupling method 4). After application of the secondary filter, no statistically significant difference remained between this method’s infested and negative control measurements, rendering it the only coupling method evaluated as unsuitable. All remaining four methods were deemed suitable for practical use. These results indicate that non-destructive coupling—including removable adhesives and compliant intermediate materials—is compatible with reliable AE detection of *H. bajulus* larval activity, while heavily damped intermediate layers (stiff cardboard combined with thin film) can attenuate the signal to the point of compromising detection.

### 3.3. Effect of Sensor-to-Larva Distance on AE Detection

**Figure 10:**
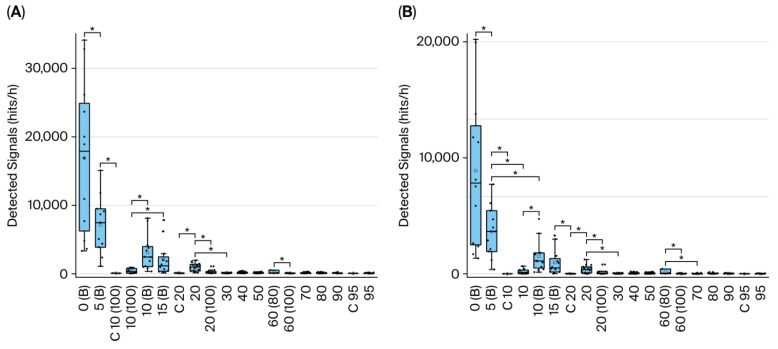
Distance measurement results with *H. bajulus* specimens and negative control specimens (C) sorted by distance; groups with no statistically significant difference summarized into one box. Numbers on the x-axis correspond to the approximate distance of the larva from the sensor in cm. (B) = measurement collected when coupling the specimen to negative control specimens; (80) = measurement collected when coupling the specimen to an 80 cm spruce lath; (100) = measurement collected when coupling the specimen to a 100 cm spruce lath. (**A**): Unfiltered data. (**B**): Filtered data. Explanation of the box plot symbols see Figure 6. The * and bar symbol on top mark experiments with significant different results. (Source: Figure 35 in Ref. [25]).

The general hypothesis that detected signal intensity decreases with increasing sensor-to-larva distance was confirmed (Figure 10). The steepest decline in hit rates occurred between direct coupling (0 cm) and the first offset distance tested (~5 cm), at which a statistically significant decrease was already observed. However, once a larva has been placed in the sample, it moves through the channel created as it feeds and can therefore no longer be pinpointed. Statistically significant differences continued to be detected with increasing distance up to approximately 30 cm. At distances beyond approximately 30 cm, measured hit rates were generally low and no longer significantly distinguishable from negative control values, indicating that reliable activity detection at these distances was not achievable under the conditions tested.

Several methodological constraints limit the generalizability of these results. In the long-distance group, the larva was housed in a separate specimen clamped to the measurement lath rather than being embedded within the lath itself; this interface between the specimen and the lath introduces an additional acoustic discontinuity, likely reducing signal transmission relative to a monolithic sample. Furthermore, the compound geometry of the test assembly (multiple wood pieces of varying dimensions) differs from the continuous solid timber that would be encountered in structural applications, where signal propagation along the grain direction could be expected to exceed 30 cm [21,22]. These constraints are, however, directly relevant to the conservation context: cultural heritage objects are frequently composite assemblies of multiple wood elements, and acoustic discontinuities at joints are a practical limitation that must be accounted for in measurement planning.

### 3.4. Verification on Structural Timber: Large-Sample Experiments

**Figure 11:**
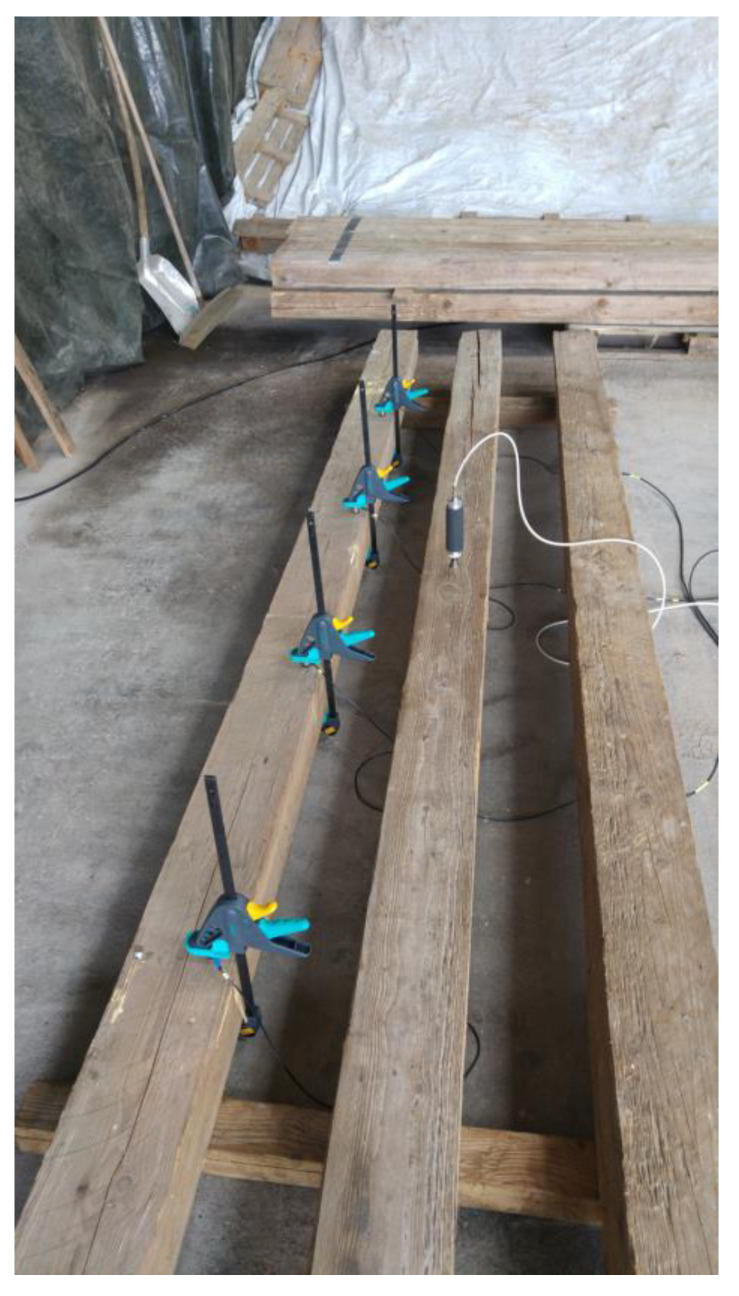
Comparative measurement with AMSY-6 measuring system and Woodworm Detector on squared timber; 4 AMSY sensors fixed with blue clamps on the left, WWD sensor with white cable in the middle. Photo: courtesy of U. Noldt.

To assess the practical applicability of AE measurement on structurally relevant timber and to evaluate whether larval infestation can be localized and approximately quantified, comparative measurements were conducted on squared pine timber sections (cross-section 160 × 120 mm^2^, length 400 cm; *n* = 14 samples) previously used as weather protection cladding and showing visible infestation signs typical for *H. bajulus* infestation. The AMSY-6 four-channel system and the Woodworm Detector (WWD, single-channel AE instrument) were operated simultaneously on the same sections to enable direct comparison of the two instrument types (Figure 11).

**Figure 12:**
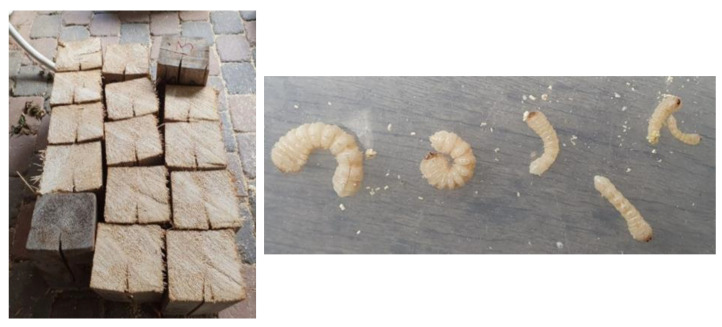
(**Left**): Overview of large timber samples and cross-cutting into 15 segments for post-measurement dissection. (**Right**): examples of large, medium, and small *H. bajulus* larvae found in segments. Photo: courtesy of U. Noldt.

Each timber section was measured in a series of sub-section positions, with sensors placed at positions corresponding to ¼ and ¾ of the beam length, for approximately 15 min per position (total: ~30 min per beam). AMSY-6 sensors were clamped laterally to the timber; the WWD sensor was mounted via a magnetic plate attached with a threaded screw. Following completion of all acoustic measurements, each timber section was cross-cut into 15 successive segments (Figure 12, left), and each segment was inspected to enumerate and size-classify the larvae found (large, medium, small; Figure 12, right). Acoustic measurements were then retroactively assigned to the segment in which the sensor had been positioned.

**Figure 13:**
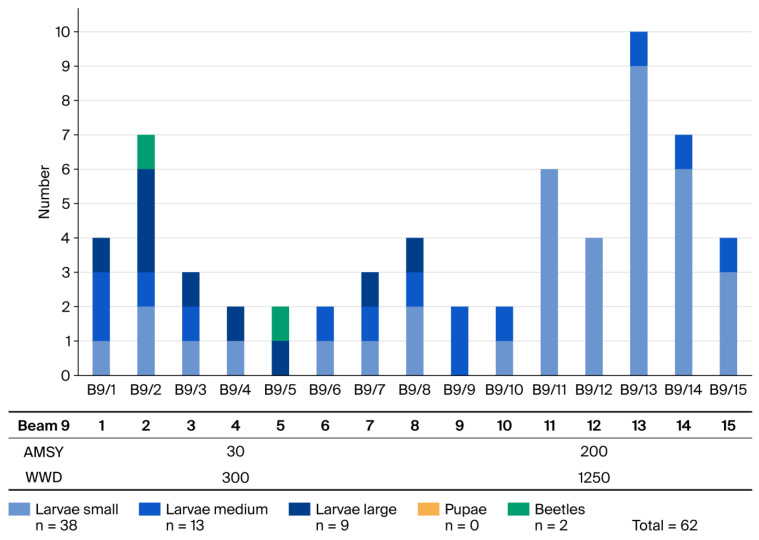
Sample 9 (high infestation level): larval count per segment (bar chart, top) and corresponding AMSY-6 and WWD pulse rates in hits/h for sensor positions in segments 4 and 12 of beam 9. Diagram: courtesy of J. Creemers. Source: Ref. [28].

**Figure 14:**
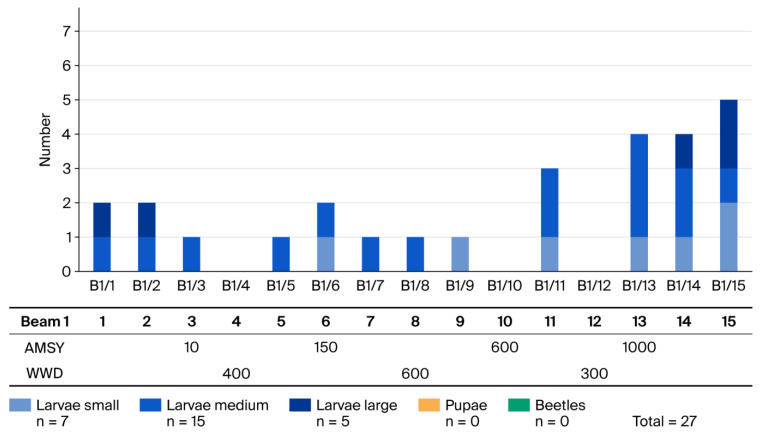
Sample 1 (medium infestation level): larval count per segment and corresponding AMSY-6 pulse rates (sensors in segments 3, 6, 10, and 13) and WWD pulse rates (sensors in segments 4, 8, and 12 of beam 1). Diagram: courtesy of J. Creemers. Source: Ref. [28].

Both the AMSY-6 and the WWD reliably detected larval activity in sections with confirmed infestation (Figure 13 and Figure 14). Hit rates, extrapolated to hits/h, showed a consistent directional relationship with the number and size of larvae in adjacent sections: sections with high larval densities produced higher hit rates; sections with low or absent larvae produced near-background rates. Approximate thresholds for activity detection under these conditions were as follows: >150 hits/h for the AMSY-6 configuration; >50 hits/h for the WWD. These values differ between instruments owing to differences in sensor positioning (sensors were not co-located but placed at approximately the same longitudinal position), frequency sensitivity range, and gain settings, which affect background noise levels differently.

Precise localization of individual larvae within sections was not achievable, as the effective sensing range of the sensors spans multiple segments simultaneously and exact range limits in structural timber dimensions cannot be determined without more systematic distance calibration. However, the results confirm that broader localization—identifying which end or quarter of a timber member contains the active infestation—is feasible with a small number of sensor positions, providing useful information for targeted inspection or intervention.

### 3.5. Field Applications

The instrumentation and protocols described above have been applied by wood protection experts in over 100 building assessments across Germany, Austria, and Switzerland, including numerous historic buildings and churches. Four representative cases are described below to illustrate the range of application contexts and diagnostic outcomes. More experiences are reported at https://insectactivitydetectionsystem.de/ (URL accessed on 20 July 2026).

For the measurements described in Section 3.5.1, Section 3.5.2 and Section 3.5.3, double-sided adhesive pads (3M Heavy-Duty, 25 mm diameter, 0.6 mm thick, Manufacturer: 3M Germany) were used. For the measurements described in Section 3.5.4, the sensor was coupled “dry” using a clamp. The distance between the sensor and interest zones was between 10 and 20 cm.

#### 3.5.1. Sacristy Cupboard Door, Church in Munich (2023)

**Figure 15:**
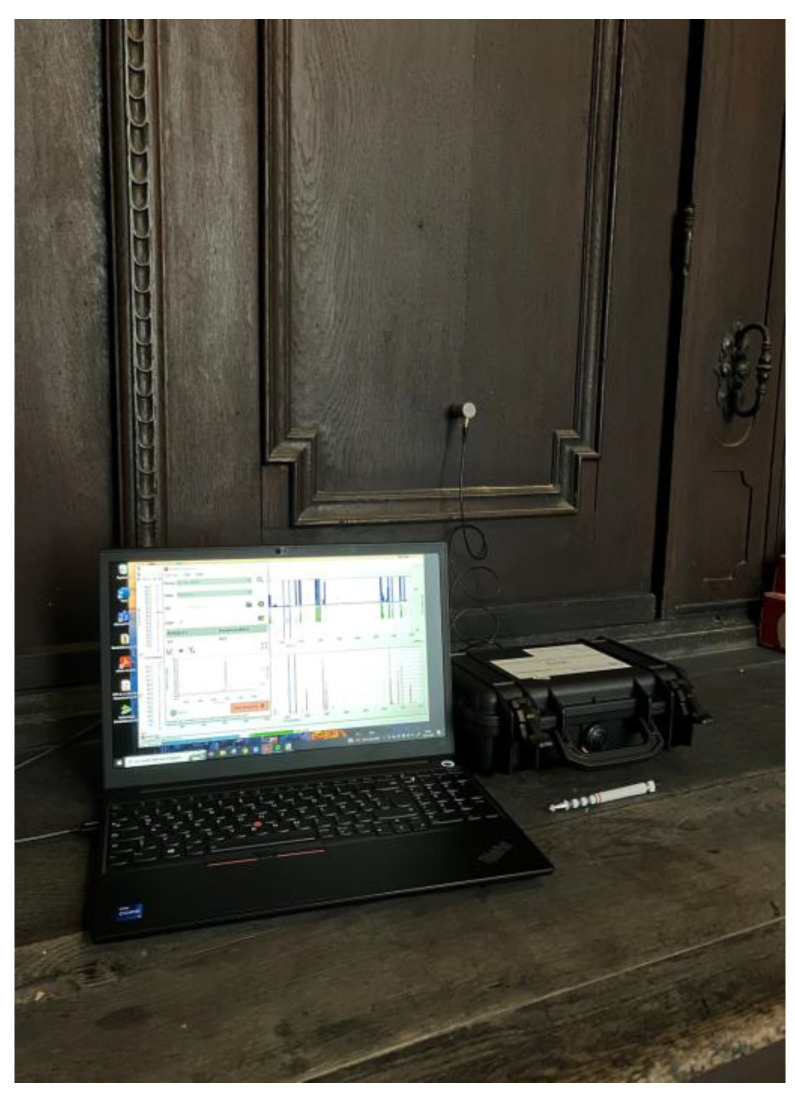
Cupboard door with sensor and IADS equipment. Photo: S. Biebl.

A cupboard door in the sacristy of a church showed frass and emergence hole patterns consistent with *Anobium punctatum* infestation. The climatic conditions showed 52% rH at 11 °C with a measured wood moisture of over mass-15%. IADS measurement with an AE sensor (frequency range 25–80 kHz) spaced 10 cm from fresh exit holes confirmed larval activity based on the detection of multiple hit clusters above the activity threshold (Figure 15). The measurement showed 3 hits per minute over a period of 41 min which indicated larval activity inside the wood. Based on this result, removal of the door by a conservator and treatment in an anoxic atmosphere (oxygen-free enclosure, approximately below 1% O_2_) was recommended and subsequently carried out.

#### 3.5.2. Door Frame, Historic Log Cabin, Garmisch (2024)

**Figure 16:**
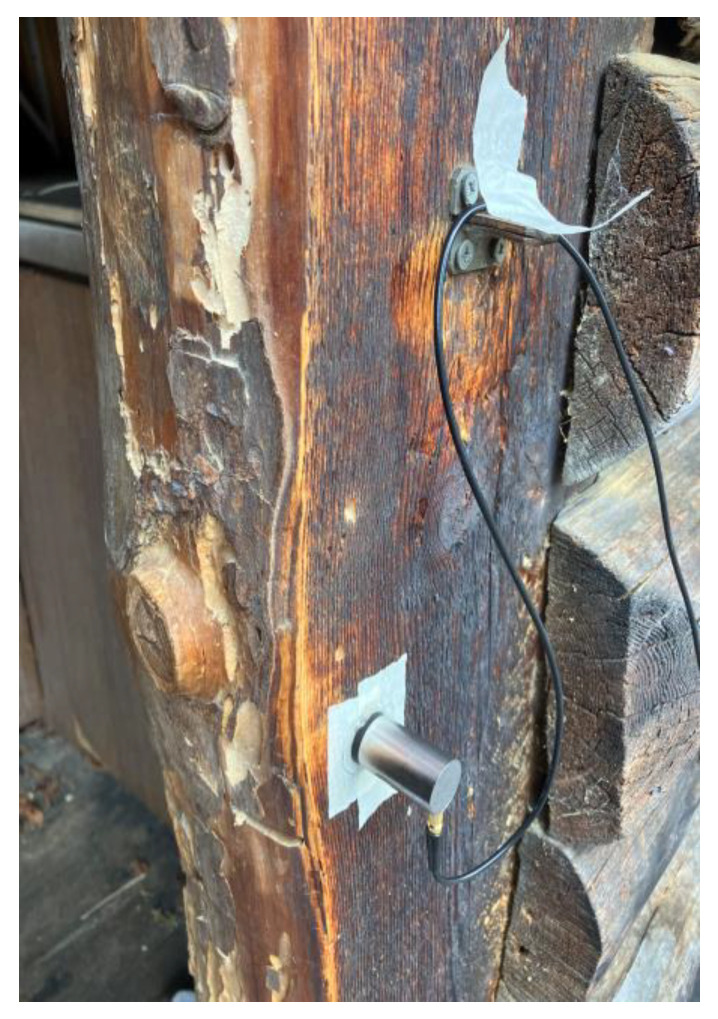
AE sensor in a historical wooden log cabin. Photo: S. Biebl.

A door frame of a historic wooden cabin was inspected due to suspected old house borer (*Hylotrupes bajulus*) infestation on the basis of exit holes and drilling dust. IADS measurement with an AE sensor (frequency range 25 kHz … 80 kHz) confirmed larval activity acoustically, corroborated by audible feeding sounds directly perceived by the expert assessor at the measurement site—providing independent verification of the acoustic instrument’s result (Figure 16). The measurement showed 101 hits per minute over a period of 6 min. which indicates high larval activity inside the wooden door frame. The high impulse rate can be attributed to larval density in the wood and the measured high outside temperatures of 28 °C. A formal expert opinion with recommendations for treatment and structural monitoring was issued to the building owner.

#### 3.5.3. Historic Staircase Cladding, Munich (2025)

**Figure 17:**
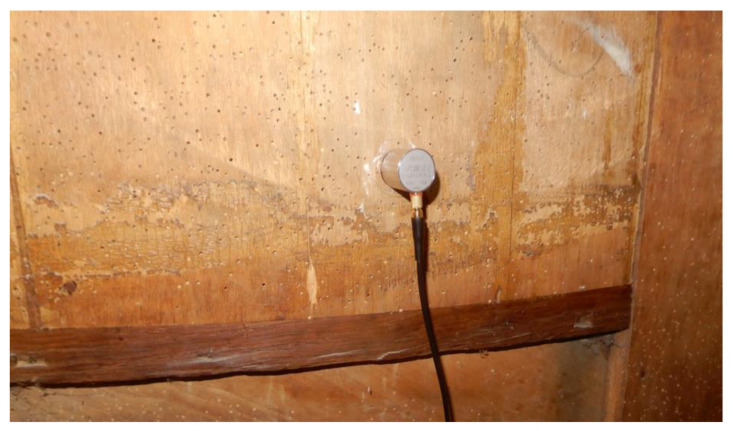
AE sensor and infestation characteristics on a side wall of a historic staircase. Photo: S. Biebl.

Multiple emergence holes and fresh frass on the side wall paneling of a historic staircase scheduled for refurbishment indicated *Anobium punctatum* infestation. IADS measurement revealed localized but active larval activity confined to a restricted area of the cladding (Figure 17). The measurement showed 4 hits/min over a period of 6 min. Despite a low pulse rate at the measuring point, an active infestation was diagnosed due to a high number of fresh exit holes and a wood moisture content of over mass-16%, at a room temperature of 20 °C and a humidity of 70% rH. Based on the measurement’s spatial localization, a targeted microwave heat treatment (minimum 55 °C for at least 60 min at the infestation site) was applied in place of a broader and more costly intervention. Without the acoustic measurement, either a more extensive treatment affecting the entire structure would have been necessary, or a multi-month visual monitoring period would have been required—both options were incompatible with the planned construction timetable. The treated area was returned to regular use following treatment.

#### 3.5.4. Winged Altar, Church in Weißenburg (2024)

**Figure 18:**
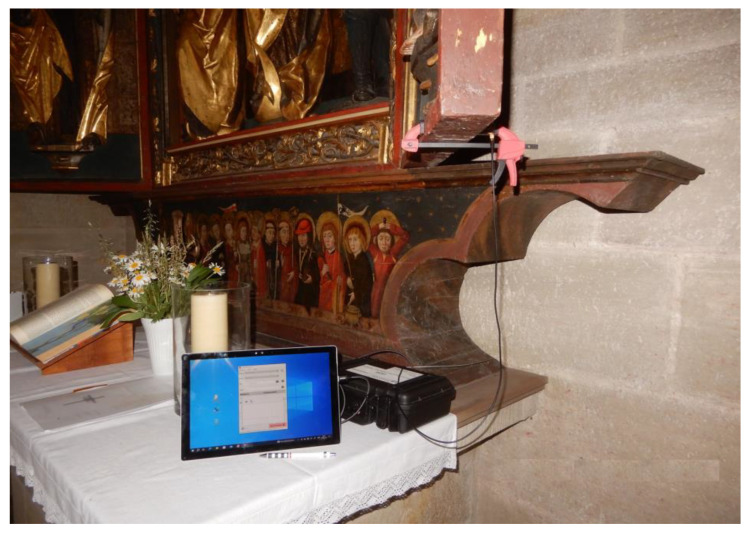
AE sensor and equipment on a wooden altar. Photo: S. Biebl.

Emergence holes and frass on a painted winged altar raised suspicion of active *Anobium punctatum* infestation. The IADS measurement with 0 hits per minute over a period of 6 min detected no larval activity above background (Figure 18). The indoor climate was recorded using a handheld meter, showing a temperature of 18.5 °C and a humidity of 55% rH. A wood moisture meter recorded a wood moisture content of 15.5 weight-%. Supplementary chemical analysis was used to identify residues of historical wood preservatives on the altar surface, indicating prior treatment. The combination of negative acoustic result and chemical analysis evidence supported the conclusion that the infestation was no longer active, and costly treatment was avoided. However, a monitoring protocol was recommended.

### 3.6. Additional Applications

Beyond the core diagnostic use case, the AE method has been successfully applied to several further specialized tasks, including the following:

- Efficacy assessment of wood preservative treatments by comparing larval hit rates before and after biocide exposure in standardized specimens, where a 5–30 min measurement period could confirm or exclude larval activity with sufficient confidence [27], Chapter AP 8.3.
- Monitoring *H. bajulus* activity in stem sections with suspected Asian longhorned beetle (*A. glabripennis*) infestation in quarantine and climate chambers with simulated day–night temperature cycling using AMSY-6, demonstrating that hit rates track simulated temperature fluctuations [27], Chapter AP 7.1.
- Sequential measurement of structural timber in a timber trade setting for rapid screening of imported goods with suspected infestation; [27], Chapter AP 8.2.
- Monitoring thermal treatment efficacy, where the characteristic pattern of initial activity increase, subsequent cracking signals during heating, and complete cessation of AE activity after cooling confirmed larval kill in a self-regulating heating cable experiment [27,34], Chapter AP 7.3.
- Successful preliminary tests on materials in a library context, infested by *Stegobium paniceum* (drugstore beetle) [35].

## 4. Discussion

### 4.1. Experimental Limitations and Interpretation

The experimental results reported here must be interpreted within the boundaries of the study design. Several limitations are noted explicitly.

Sample sizes in the small-specimen experiments were modest (*n* = 20 infested specimens for the temperature and coupling experiments; *n* = 12 + 11 for the two distance groups), and in the temperature experiment only a single negative control specimen was included. While the directional results are clear and internally consistent, the small negative control group in the temperature experiment precludes rigorous statistical comparison with controls for the warm vs. cold conditions, and the absence of replication of the control limits confidence in the specificity of the threshold. Future experiments should include a substantially larger number of negative controls (recommended minimum *n* = 5–10 per condition).

All small-specimen experiments used standardized specimens of a single, small size (50 × 25 × 15 mm^3^). This format maximizes experimental control and reproducibility but does not replicate the acoustic propagation conditions encountered in large structural timbers or composite cultural heritage objects. Signal attenuation characteristics, effective detection range, and the relative impact of background noise may all differ substantially in field conditions. The large-sample experiments (Section 3.4) partially address this gap, but a more systematic experimental investigation of range and attenuation in structural elements of varying dimensions remains a priority for future research.

In the distance experiments, the compound geometry of the test assembly (a separate specimen clamped to a measurement piece, rather than a larva embedded within the measurement piece itself) constitutes an additional acoustic interface. Although a similar situation—a measurement being conducted on a piece of wood that is directly joined to an infested piece instead of a single piece of solid wood—may be present in real infestation scenarios (especially in the case of cultural artifacts), this poses suboptimal signal transmission conditions for the sensor. This likely led to a conservative (lower-bound) estimate of the effective detection range. For more robust estimations of the effective sensor range under different conditions that could occur in practice, systematic distance experiments using monolithic solid timber with embedded larvae at defined positions should be a priority for future work.

Finally, since the field tests were conducted under real practical conditions, the factors influencing the measurement results are numerous and highly complex. Therefore, the resulting measurement data must be interpreted with these unknown details in mind and cannot provide the same information and statistical power as the more systematic experiments. Nevertheless, these field tests remain relevant for testing the general practical feasibility of this technology.

### 4.2. Practical Implications and Expert-Dependency

The results collectively support the use of AE measurements as a reliable, non-destructive tool for helping to confirm or rule out active wood-boring insect infestations in a wide range of contexts—wooden structures, cultural heritage objects, timber trade, and controlled biocide testing. The field cases presented illustrate the practical decision-making value of the method: treatment avoidance where infestation is acoustically confirmed as inactive (Section 3.5.4); targeted rather than extensive treatment where activity is spatially confined (Section 3.5.3); and corroboration of acoustic results by independent evidence (direct auditory perception, Section 3.5.2).

However, it is essential to note that AE measurements do not replace but support expert knowledge. The accurate interpretation of results requires a background in wood biology, species identification, infestation assessment, and wood conservation, and an understanding of the physical constraints of the measurement (temperature, specimen geometry, background noise, and coupling quality). In particular, a negative result—no AE activity detected above threshold—should never be interpreted as proof of absence without a measurement of sufficient duration (which may be species-dependent), adequate coupling confirmed using PLB testing, and a measurement temperature above the lower activity threshold. These prerequisites are readily met by trained wood protection experts but may not be by untrained users.

A key feature of the sensor technology we use is that it is only sensitive to sounds in a frequency range outside the range which a human can hear, which means we measure only beyond approx. 20 kHz. Therefore, low-frequency interference from structure-borne sound (e.g., footsteps, slamming doors) or airborne sound (e.g., voices, machinery) is practically ruled out. This is checked once again during the pencil lead test. Interference caused by thermal expansion is highly unlikely at temperatures at which larvae are active. Larval activity is also characterized by cascade-like sequences of hits, see Figure 4c. If, based on this experience, noise cannot be distinguished from larval activity, a measurement is not possible.

### 4.3. Future Directions

Several research priorities are identified on the basis of the present results and their limitations.

First, systematic distance and attenuation experiments using monolithic timber of varying dimensions and species, with larvae of known mass, would enable evidence-based sensor spacing recommendations for structural applications.

Second, extended-duration, large-sample experiments specifically targeting *A. punctatum*—and other practically relevant species including *Xestobium rufovillosum*, *Lyctus brunneus*, *Anoplophora glabripennis,* and termites (*Reticulitermes* spp., whose expanding European range under climate change increases their relevance [36,37])—are necessary to establish species-specific measurement duration recommendations and detection thresholds.

Third, the integration of machine learning for automated hit classification [24] or species identification from frequency-domain signal features represents a technically promising direction, but the training data requirements are substantial and must account for the combinatorial complexity of instrument settings, specimen geometry, wood species, larval species, developmental stage, and temperature.

Fourth, the development and validation of standardized measurement and reporting protocols—analogous to standards for related NDT methods—would significantly advance the practical adoption of AE insect detection in wood protection and conservation practice.

## 5. Conclusions

This article presents a systematic, integrated account of acoustic emission (AE) measurements for the detection of wood-boring insect larval activity, from physical principles through controlled laboratory experiments to field applications in structural and cultural heritage contexts. The main conclusions are as follows:

1. AE measurements can be used to reliably detect *H. bajulus* larval activity in standardized small specimens at hit rates clearly distinguishable from negative controls under all five coupling methods tested, except the heavily damped cardboard/thin-film combination.
2. *H. bajulus* specimens stored at 10 °C can be measured directly without a warm-up phase; hit rates increase after room-temperature acclimatization, consistent with ectothermic temperature dependence. A provisional lower measurement threshold of 10 °C is recommended.
3. A reliable AE detection range in the compound-geometry distance setup was approximately 30 cm; results in monolithic structural timber are expected to exceed this.
4. A measurement duration of 30 min is usually sufficient for *H. bajulus* under favorable conditions (substrate, temperature, and relative humidity) for this species.
5. AE measurements on large structural timber enabled qualitative infestation localization and confirmed detection performances that were comparable between the AMSY-6 and Woodworm Detector systems.
6. Field applications in historic buildings and on cultural heritage objects demonstrate the method’s practical value: enabling targeted treatment decisions, avoiding unnecessary intervention, and documenting negative results with confidence when measurement conditions are met.
7. The method requires interpretive expertise in wood biology; infestation assessment; and, in the case of cultural artifacts, conservation expertise. AE measurement is a decision-support tool, not a substitute for expert judgment.

## Data Availability

The results in the Section 3.1 “Temperature Effect on AE Detection of *H. bajulus* Larvae”, Section 3.2 “Effect of Sensor Coupling Method on AE Detection”, and Section 3.3 “Effect of Sensor-to-Larva Distance on AE Detection” are documented in the thesis “Limmer, L.A. Untersuchungen zu Anwendungsmöglichkeiten des IADS in der Restaurierungspraxis (Investigations on applications of the IADS in practical restauration). Master Thesis, HAWK Hochschule für angewandte Wissenschaft und Kunst—Fachhochschule Hildesheim/Holzminden/Göttingen, Hildesheim, 2024.”, cited as Ref. [25] which is available upon reasonable request, to be directed to Constanze Messal, mailto: constanze.messal@hawk.de. The results in the Section 3.4 “Verification with large samples” and Section 3.6 “Further applications” are described more thoroughly in the report “Plinke, B. InsectDetect: Detektion aktiver Schadinsekten im Holzhandel (Detection of active wood-destroying insects in wood trade); Schlussbericht 22WK412101; Fraunhofer WKI: Braunschweig, 2021”, cited as Ref. [27] and available online here https://www.fnr.de/ftp/pdf/berichte/22WK412101.pdf (URL accessed on 20 July 2026). The expert analyses for results in the Section 3.5 “Applications in buildings and art objects” were carried out by the Ingenieurbüro für Holzschutz, Benedikbeuern, Germany. Details will be given upon reasonable request to be directed to Stephan Biebl, mailto: info@holzwurmfluesterer.de. The data collected during the investigations are not available in standard data formats and are therefore not freely available. Detailed evaluations and data on measurement conditions and settings can be found in the publications in which the authors were involved, and can be made available on request from the authors.

## Abbreviations

The following abbreviations are used in this manuscript:

AE	Acoustic emission
ALB	Asian longhorn beetle (*Anoplophora glabripennis*)
AMSY-6	Acoustic emission measurement system, type AMSY-6 (Vallen Systeme GmbH)
°C	degrees Celsius
EWGAE	European Working Group on Acoustic Emission
HAWK	Hochschule für Angewandte Wissenschaft und Kunst, Hildesheim
HNS	Hsu–Nielsen source
IADS	Insect Activity Detection System
IPM	Integrated Pest Management
IRG	International Research Group on Wood Protection
MPA	Materialprüfanstalt Eberswalde
NDT	Non-destructive testing
PLB	Pencil lead break (Hsu–Nielsen source test)
rH	relative air humidity
SHM	Structural health monitoring
SQL	Structured query language
WKI	Wilhelm-Klauditz-Institut, Fraunhofer Institut für Holzforschung, Braunschweig
WWD	Woodworm Detector
